# In-Vitro Cytotoxicity Study: Cell Viability and Cell Morphology of Carbon Nanofibrous Scaffold/Hydroxyapatite Nanocomposites

**DOI:** 10.3390/molecules26061552

**Published:** 2021-03-11

**Authors:** Asmaa M. Abd El-Aziz, Azza El-Maghraby, Andrea Ewald, Sherif H. Kandil

**Affiliations:** 1 Fabrication Technology Research Department, Advanced Technology and New Materials Research Institute, City for Scientific Research and Technological Applications, Alexandria 23713, Egypt; chemist_asmaa25@yahoo.com; 2 Department of Materials Science, Institute of Graduate Studies and Research, Alexandria University, Alexandria 21526, Egypt; s.kandel@usa.net; 3 Department of Functional Materials in Medicine and Dentistry, University Hospital Würzburg Center for Dental, Würzburg University, 97070 Würzburg, Germany; andrea-ewald@fmz.uni-wuerzburg.de

**Keywords:** HA modifiedCNF membranes, cytotoxicity, WST test, cell counting, cell viability, cell morphology

## Abstract

Electrospun carbon nanofibers (CNFs), which were modified with hydroxyapatite, were fabricated to be used as a substrate for bone cell proliferation. The CNFs were derived from electrospun polyacrylonitrile (PAN) nanofibers after two steps of heat treatment: stabilization and carbonization. Carbon nanofibrous (CNF)/hydroxyapatite (HA) nanocomposites were prepared by two different methods; one of them being modification during electrospinning (CNF-8HA) and the second method being hydrothermal modification after carbonization (CNF-8HA; hydrothermally) to be used as a platform for bone tissue engineering. The biological investigations were performed using in-vitro cell counting, WST cell viability and cell morphology after three and seven days. L929 mouse fibroblasts were found to be more viable on the hydrothermally-modified CNF scaffolds than on the unmodified CNF scaffolds. The biological characterizations of the synthesized CNF/HA nanofibrous composites indicated higher capability of bone regeneration.

## 1. Introduction

To regulate the actions of cells, tissue engineering (TE) uses physical, chemical, biological, and engineering processes. More specifically, the field of regenerative medicine (RM) is defined as the application of TE and is related to the principles of biology and engineering that restore the structure and function of damaged tissues and organs [1]. This involves not only in vivo, but also in vitro functional tissue generation ideal for various purposes, such as drug testing, disease models, including cell/tissue/organ one-chip approaches [2]. Cell activity, including cell adhesion, morphology, proliferation, and differentiation, is influenced by the surface topography of the scaffolds [3].

Techniques such as soft lithography, photochemistry, and inkjet printing have been introduced to produce a surface with a specific topography. Among them, electrospinning is a simple and flexible method of modifying surface topography by adapting a different grounded platform to establish adjustable nanofiber orientation [4]. The behavior of the cells has also been found to be modulated by their microenvironments, such as soluble factors, neighboring cells, and extracellular matrix (ECM) composition for cell adhesion [3].

Collagen and some polymers, such as poly(glycolic acid) (PGA), poly(l-lactic acid) (PLLA), poly(d,l-lactic-co-glycolic acid) (PLGA) copolymers, poly(ε-caprolactone) (PCL), ethylene glycol-based copolymers, and polyacrylonitrile (PAN) [5,6,7], are different examples of scaffolding materials that suit the intended applications. PAN is manufactured in the form of gels, bulk porous scaffolds, and nanofibers. Among polymers, due to its high mechanical properties and strong solvent resistance, PAN is an important polymer material, which is selected for filtration and composite materials [8].

PAN nanofibersare obtained by spinning, which has become the most widely used method involving spinning of polymeric precursor fibers and thermal treatment (stabilization and carbonization) to fully convert them to CNFs [9]. To stabilize scaffolds, CNFs can also be applied and provide conductivity and favorable support for different cell events and tissue regeneration. CNFs, which lack functional groups on their surfaces, are essentially bio inert. To functionalize CNFs, heavy acid treatment has been used. Bioceramic components are typically considered great in making scaffolds with enhanced biocompatibility for bone regeneration [10].

Mechanical forces modify cell surface charges by regulating cell proliferation and differentiation by controlling ion channels and altering the cytoskeleton structure by generating electrical signals [10,11].

The idea is to develop physicochemical relations between hydroxyapatite (HA) and the bone tissue surrounding it, facilitating the integration and development of the tissue. The contact that can occur at the interface with a living tissue is one of the most significant aspects of the application of these materials both in terms of toxicity, such as dissolution, and the active roles that facilitate the creation of new bones [12].

Hydroxyapatite is composed predominantly of microscopic calcium phosphate crystals, whose chemical formula is Ca_10_(PO_4_)_6_(OH)_2_ [10,11]. We prepared CNFs that had been functionalized by HA in the current work to increase their bioactivity and biocompatibility and to mimic the composite structure of collagen fiber/HA in the natural bone. The structural and biological characterizations of the composites prepared as shown in Table 1, were also studied. Besides, the invitro biocompatibility of fibroblasts of L929 was studied. The morphological changes, surface coverage, viability, and cell proliferation on the scaffolds were studied.

## 2. Materials and Methods

### 2.1. Materials

Polyacrylonitrile (PAN) was purchased from Sigma-Aldrich (Sigma-Aldrich, St. Louis, MO, USA) (average molecular weight =150,000 g/mol, d = 1.184 g/mL at 25 °C). N,N-dimethylformamide (C_3_H_7_NO) was obtained from El-Goumhouria Company (Cairo, Egypt), (molecular weight = 73.10, minimum assay (GC), 99.0%). L929 mouse fibroblasts (ATCC CRL-6364), and cell culture media (4 mM glutamine, 10% fetal bovine serum, and 100 units/µL of penicillin/streptomycin) were purchased from Invitrogen Life Technologies (Karlsruhe, Germany). Live/Dead^®^ Viability/Cytotoxicity assay kitswere produced by Life Technologies Company. Accutase, hexamethyldisilazane (HMDS) and 25% glutaraldehyde were purchased from Sigma-Aldrich (Sigma-Aldrich, St. Louis, MO, USA).

### 2.2. Samples Preparation and Characterization

**First type of scaffolds**: The polymeric nanofibers were prepared using 5 wt.% PAN solutions, PAN/0HA and PAN/8% HA, and were fed with a constant rate of 0.5 mL × h^−1^ into a syringe needle using a syringe pump. By applying a specified voltage of 30 kV, PAN solutions were electrospun on an aluminum foil by a drum collector with a fixed velocity, located at a distance of 10 cm from the needle. The PAN nanofibers (NFs) were collected after 24 h of continuous electrospinning at ambient temperature and ambient relative humidity of 70–75%.

The prepared polymeric nanofibrous membranes were PAN and PAN/8HA thermally treated by stabilization and carbonization processes to prepare carbon nanofibrous membranes, CNF and CNF-8HA [13].

**Second type of scaffolds**: CNFs produced in the first step were hydrothermally functionalized with HA. The naturally annealed HA, which was prepared in our laboratory, was dispersed in distilled water with 8 wt.% HA. The CNF membranes were cut dimensionally (30 × 20 mm2) and immersed in the as-obtained suspension or solution (40 mL). The mixture was placed into an autoclave and hydrothermally treated for 90 min at 130 °C. The resulting samples were left to dry at room temperature and were then placed in a vacuum oven (10 mbar) at 40 °C for 72 h prior to further processing [14].

The CNF precursor samples and HA-modified samples were coated with gold and investigated microscopically using a scanning electron microscope (SEM JEOL JSM 6360LA, Japan), with electron dispersive X-ray (EDX) for each sample (*n* = 3).Microscopic size of nanofibers was characterized by transmission electron microscopy (JEOL 2100 PLUS, Japan).Hydrophilicity of the prepared scaffolds was studied by static contact angle (Right/left) RL drop phase (water), external phase (air), and solid phase (polyethylene).

### 2.3. Characterizations of the Biological Properties

Pure carbon nanofibrous (CNF) scaffolds (with a thickness of 5.0 mm) were used as a control sample to verify the cytocompatibility of the CNFs modified with hydroxyapatite (HA). Prior to biological experiments, the samples were sterilized with 70% ethanol for 30 min [14,15], washed three times with sterile PBS, subsequently immersed in a culture medium for 30 min, and then placed in a 96-well plate (Nunc, Wiesbaden, Germany) for cell culture experiments [16,17]. L929 mouse fibroblasts (ATCC CRL-6364) were used to assess the biocompatibility of the nanofibers, and 5 × 10^4^ cells/mL were cultured for each 6.0 mm rounded sample (*n* = 3). The samples covered the bottom of each well, the cells were enriched with the Dulbecco’s modified Eagle’s medium supplemented with 4 mM glutamine, 10% fetal bovine serum, and 100 units/µL penicillin/streptomycin (Invitrogen Life Technologies, Karlsruhe, Germany) and incubated at 37 °C in a humidified atmosphere with 5% CO_2_ [18].

The cell metabolic activity was analyzed using the WST assay with the WST-1 reagent (Roche Diagnostics, Mannheim, Germany). TheWST-1 molecule is metabolized by an enzyme of the respiratory chain in mitochondria into a yellowish Formazan derivative and is therefore used as an indicator of ATP production. Higher absorption indicated higher cell metabolic activity. After incubating the cells for 30 min with the WST reagent diluted 1:10 in the DMEM at 37 °C, the absorption of the supernatant was quantified with a spectrophotometer at λ = 450 nm (Tecan, Crailsheim, Germany) [19].

The cell viability percentage was calculated using the following equation:

(1) $$\mathrm{Cell}\;\mathrm{viability}\;\%=\frac{\left(A\;\exp-A\;\mathrm{control}\right)}{\left(A\;\mathrm{positive}-A\;\mathrm{control}\right)}$$

where A exp is the value of the experimental absorbance of the test sample (nanofibrous scaffold + cell), A control is the value of the blank sample absorbance (media without cells), and A positive is the value of the positive control absorbance (media + cells).

Cell proliferation was analyzed by electronic cell counting using a CASY 1 TTC cell analyzer (Scharfe System, Reutlingen, Germany). The cells were detached from the surface of scaffolds by incubation in Accutase for 10 min at 37 °C. Then, the reaction was stopped, the medium was added, and the cell suspension was counted [5]. The cell viability was measured using a Live/Dead^®^ Viability/Cytotoxicity assay kit (Life Technologies, Thermao Fisher Scientific, USA) after three and seven days in the culture.

The culture medium was aspirated and replaced with PBS for 5 min. After removing PBS, the reagent was added to each construct. The reagent was made by mixing dye calceinAM (2.5 μL), ethidium homodimer-1 (10 μL), and PBS (5 mL). The constructs were incubated in the reagent in the dark for 20 min and then rinsed in PBS for 5 min. The constructs were then observed under an inverted fluorescence microscope (AXIO Imager M1; Zeiss, Oberkochen, Germany) and visualized. The green fluorescence indicated the live cells and the red fluorescence indicated the dead cells using two different filters [20].

Finally, for studying of cell morphology, the CNF scaffolds were washed with PBS twice and then fixed in 6.0 *v*/*v* % glutaraldehyde/PBS for 15 min at 4 °C. Following five rinses with PBS for 5 min at 4 °C each, the samples were dehydrated through a series of graded ethanol solutions (30, 50, 70, 90, 100% *v*/*v*) at room temperature [21]. Subsequently, the samples of the nanofibrous mats were treated with hexamethyldisilazane (HMDS), kept in a fume hood for air drying overnight, and then coated with platinum for the observation of cell morphology by scanning electron microscopy (Zeiss Crossbeam) [17].

### 2.4. Statistical Analysis

The significant differences between the data were analyzed using a one-way ANOVA test. Thevalue of *p* < 0.05 was considered to be significant.

## 3. Results and Discussion

**Figure 1:**
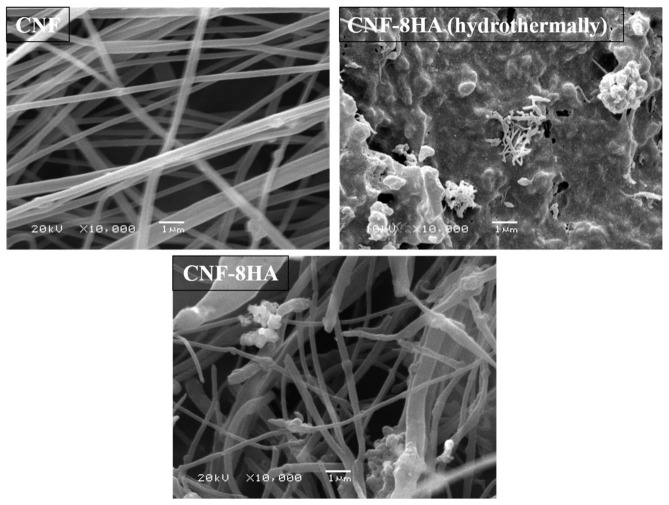
SEM images of the prepared CNF membranes.

The microscopic and spectroscopic methods revealed the surface morphology of the prepared membranes. Figure 1 shows the SEM images of CNF, CNF/8HA, and CNF/8HA (H). The images showed the surface roughness of the CNF/HA-modified membranes as compared to the unmodified ones. Rough surface is favorable for cell growth when it comes in contact with a living system in tissue engineering for both invitro and invivo studies [13]. More SEM images of nanofibrous membranes are provided as supporting data (Figure S1). Table 2 displays the elemental analysis of the modified nanofibrous samples and shows that the ratio between Ca and P is ~0.4 and 1.98 for CNF/8HA and CNF/8HA (H), respectively. The hydrothermally prepared nanofibrous sheet has a higher ratio of HA that improves surface hydrophilicity and stimulates osteogenic properties [21].

**Figure 2:**
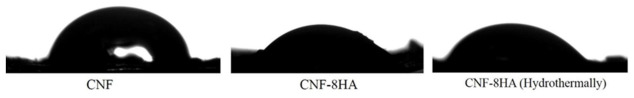
Contact angle of the prepared carbon nanofibrous scaffoldswas measured (n = 5) for each sample. The contact angles were 76 ± 0.05°, 53 ± 0.19°, 49 ± 2° for CNF, CNF-8HA, and CNF-8HA (H).

**Figure 3:**
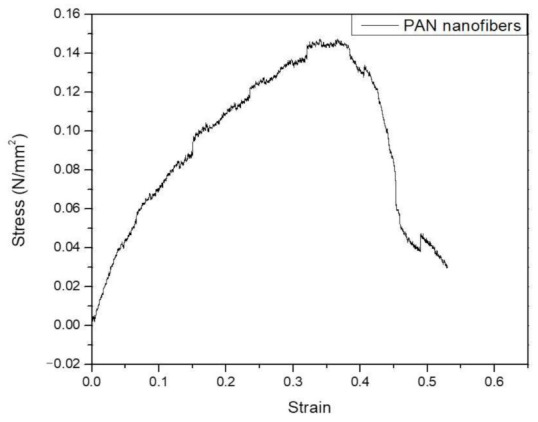
Stress–strain curve of the PAN nanofibrous membrane.

For the wettability of the prepared membranes, the static contact angle of the prepared samples showed that the contact angle of the composite scaffolds was more hydrophilic than of the pure sample as shown in Figure 2. According to our previous studies, hydrophilic surfaces increase cell adhesion in the initial step of cell culture, and cells proliferate on rough surfaces [21,22]. Polyacrylonitrile-based fibers have a more complex structure and consist of many tubular elements combined into a three-dimensional structure. Such a structure can and does provide high strength due to the overlap of tubular elements, but its high stiffness is difficult to preserve solely by in-creasing structure density and decreasing the total pore volume [23,24]. Figure 3 shows the stress–strain curve of PAN nanofibers and reveals thatthe tensile modulus of the polymeric nanofiber membrane without HA modification is equal to 0.408 ± 0.09 MPa. This value is suitable for stimulation of the periosteal membrane of the natural bone.

**Figure 4:**
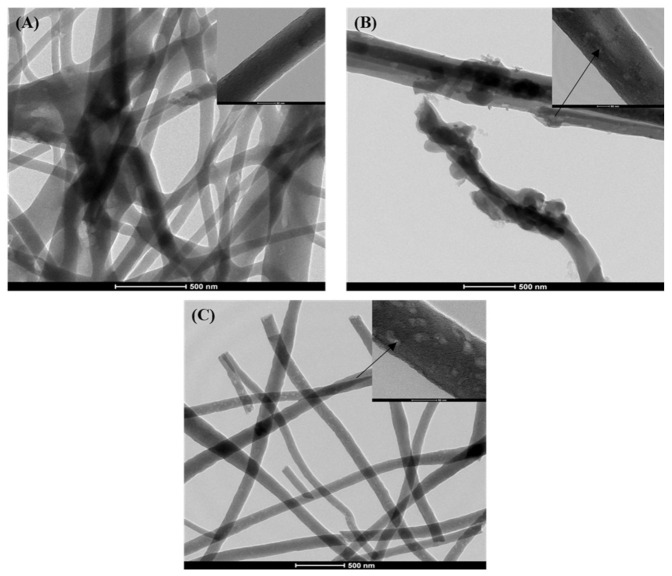
TEM images of the prepared nanofibrous samples (scale bar, 500 nm). (**A**): CNFs, (**B**): CNFs/8HA, and (**C**): CNFs/8HA (hydrothermally).Each image has a highly magnified image in the top-right corner (scale bar, 50 nm). The pointing arrows indicate the HA particles.

Figure 4 shows the transmission electron microscopy (TEM) images of the prepared samples that refer to HA nanoparticles in the CNF. The images show that the CNF appears as a nanofibrous web and the individual nanofiber has a diameter of 110 ± 28 nm. The pictures show that small-sized HA nanoparticles indicated with arrows were located inside the nanofibers and the larger ones remained partially captured on the nanofiber surfaces [12,23].

**Figure 5:**
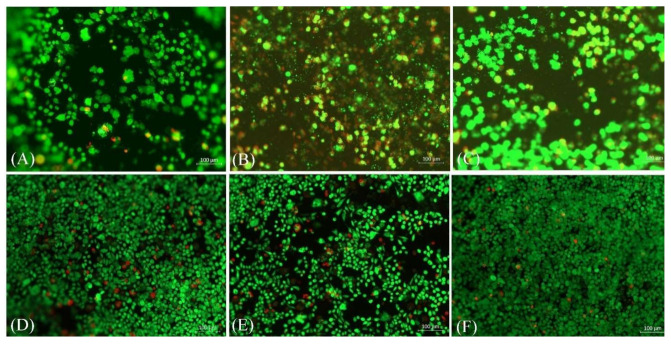
Live/dead staining images of the fibroblast cells grown on the prepared samples (CNF, CNF/8HA, and CNF/8HA (hydrothermally)after three days incubation(**A**–**C**)and after seven days of incubation (**D**–**F**), respectively. Live cells are shown in green and dead cells are red.

**Figure 6:**
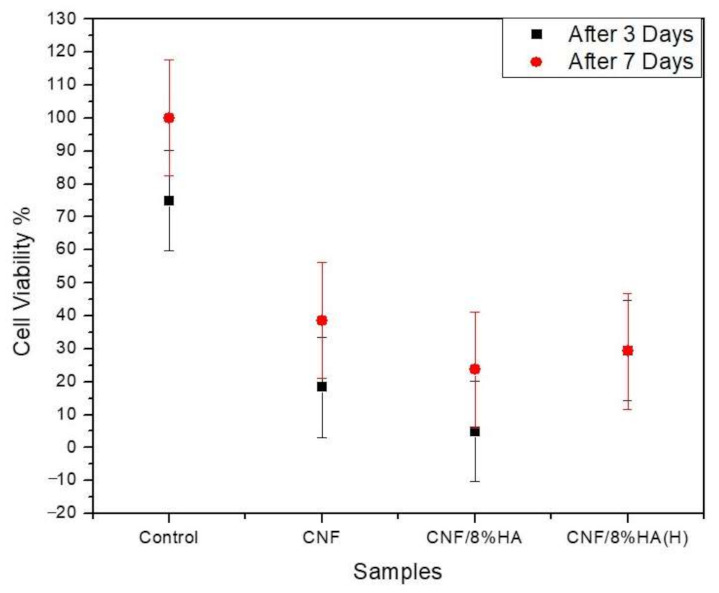
Proliferation of fibroblast cells on the prepared samples (CNF, CNF/8HA, and CNF/8HA (H) in compare with the positive control (without sample).

For the biological studies, L929 mouse fibroblast cells were seeded on the scaffolds and cultured for threeandseven days. After the specified time, a life/dead staining viability test was performed. Figure 5 shows that the presence of HA reduced the number of red (dead) cells when compared with the pure samples. The results revealed that the hydrothermally prepared samples were the most durable and the cells were interconnected with good proliferation; these results were also verified by WST viability results that are shown in Figure 6. The WST test showed that the cell viability percentage increased by 38% for CNF-8HA (H) when compared to the pure CNF membrane after three days of cell culture. Besides, the cell viability percentage after seven days decreased which were calculated by Equation (1), unlike in the pure CNF membrane, as shown in Table 3. These results may have taken place due to the confluent surface of the modified membranes.

**Figure 7:**
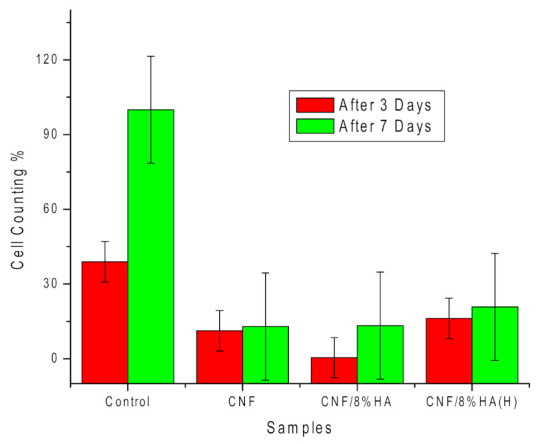
Number of cells attached to the prepared samples (CNF, CNF/8HA, and CNF/8HA (hydrothermally)) in comparison with the control (no sample) after three days (red columns) and seven days (green columns).

Figure 7 shows that the number of cells increased continuously over seven days of culture on the CNF and on the modified CNF/HA scaffolds. For the CNF/8HA sample, the number of cells increased 34 times after three days of culturing. In the case of CNF/8HA (H), the HA greatly increased the L929 cell proliferation, as the cell number on the surface was sufficiently high on the first day and a slight increase was noted after seven days Figure 7 reveals that the cell count seems to change as follows independently of the cell culture time: CNF/8HA < CNF < CNF/8HA (H).

More studies, as well as our study, have shown that there are ambiguous descriptions in regard to the effects of HA on the proliferation of cells on scaffolds [21].

**Figure 8:**
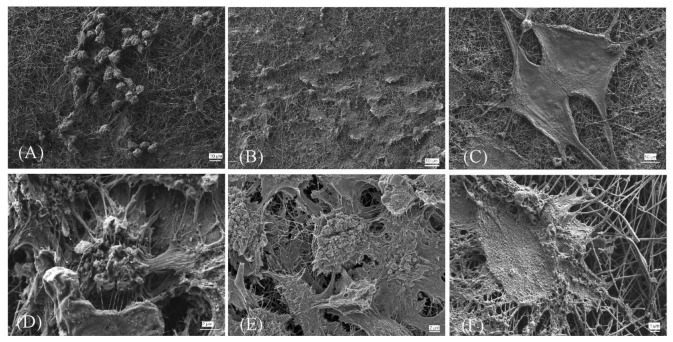
SEM images of the fibroblast cells grown on the prepared samples (CNF, CNF/8HA, and CNF/8HA (hydrothermally)) after seven days with low magnification (**A**–**C**) and high magnification (**D**–**F**).

The SEM images in Figure 8 show the morphology of the cells as it appeared on the prepared nanofibrous scaffolds after seven days; the cells formed confluent layers on both modified samples, especially on CNF/8HA (H) [3]. The microstructure of the cells that were observed by SEM gives more information about cell–matrix contacts. Those cells grew on CNF scaffolds elongated along the fibers with flattened morphology and had less clear borders that appeared between the cells and nanofibers. Furthermore, there wasstrong contact between cells with each other that was shown by cellular roots. The adhesion of the cell membrane with the nanofibers via HA is vital to transduce physical external stimuli of the cells and have a substantial influence on cellular behavior. The scaffolds which were modified with HA had a high number of hydroxyl functional groups that promote cell attachment and also cell spreading [25,26,27]. Moreover, the cells were observed to be spanning the gaps between the electrospun fibers, as indicated by the flattened and spreading morphology covering the surface [21]. Based on these in-vitro results, the CNF composite nanofibrous scaffolds that were developed in this study are considered to recruit favorable adhesion and growth of fibroblasts, as well as differentiation to osteoblasts to simulate periosteal activity for bone regeneration.

## 4. Conclusions

In addition to the agglomeration of HA that appeared as white dots on the fiber threads in the case of fiber threads, the functionalization of CNFs with hydroxyapatite (HA) did not induce any change in the average diameter of the fibers. With various sizes of HA nanoparticles, the surface of CNF/8HA (H) was rough.The surface hydrophilicity and wettability of the scaffold were enhanced by the inclusion of HA on the surface of CNF/8HA (H). In addition, cell adhesion and proliferation were enhanced by the structure of nanofibers.

The L929 fibroblast cells were well-attached and distributed on all the prepared scaffolds. In comparison with the other samples, cell interconnectivity and activity on CNF/8HA (H) were increased. Finally, the results obtained reveal that the nanofibrous carbon scaffolds generate a strong scaffold to be used for bone regeneration. As a continuation of this work, experiments on the osteogenic differentiation of the prepared scaffolds are required.

## Figures and Tables

**Table 1:** Scaffold codes with the certain method of preparation.

Scaffold Codes	Method
CNF	Without modification
CNF-8HA	Modification before electrospinning
CNF-8HA (H)	Modification after electrospinning (hydrothermal)

**Table 2:** Elemental analysis (EDX) of the modified carbon nanofibrous samples.

Sample	CNF-8HA	CNF-8HA (Hydrothermally)
**C**	95.35	54.97
**Ca**	1.3	14.58
**P**	3.29	7.33

**Table 3:** Cell viability percentage of the prepared samples after three and seven days.

Cell Viability %	CNF	CNF/HA	CNF/HA (H)
After three days	18.3 ± 10	4.8 ± 15	29.46 ± 15
After seven days	38.6 ± 17	23.7 ± 10	29.3 ± 9

## Data Availability

Data is contained within the article or supplementary material.

## Supplementary Materials

The following are available online, Figure S1: SEM images of the prepared samples, and the (CNF-8HA (H)) has high magnified image on the top right of the low magnified image (20000x).

## Sample Availability

Samples of the compounds are available from the authors.
