# Supplementary material for: In-Vitro Cytotoxicity Study: Cell Viability and Cell Morphology of Carbon Nanofibrous Scaffold/Hydroxyapatite Nanocomposites
Source: Molecules. 2021 Mar 11;26(6):1552. doi: 10.3390/molecules26061552 (PMC7999924; doi:10.3390/molecules26061552)
Supplement: Supplementary file 1 [file molecules-26-01552-s001.pdf]

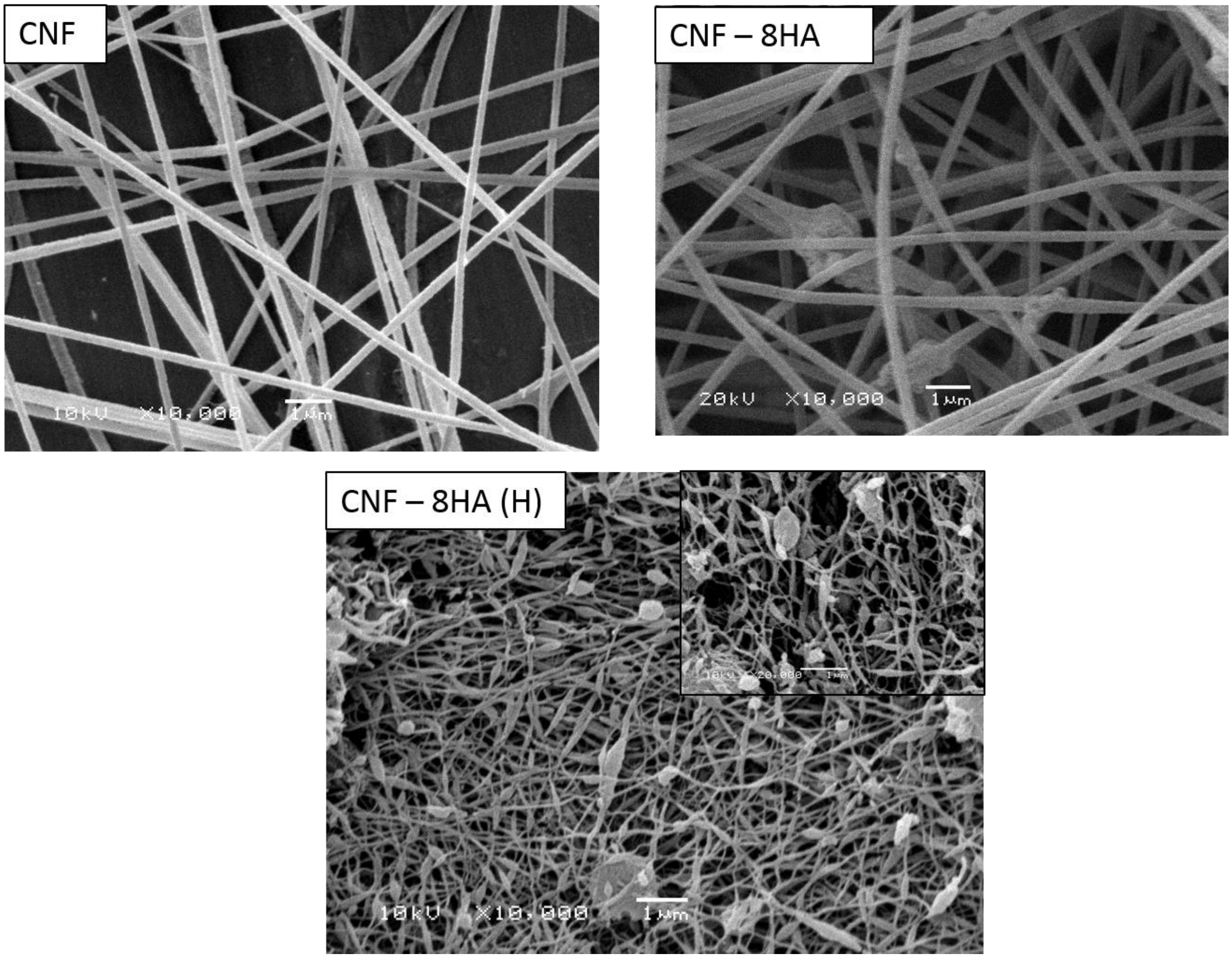


**Figure S1.** SEM images of the prepared samples, and the (CNF-8HA (H)) has high magnified image on the top right of the low magnified image (20000x).
